# Sexual dysfunction prevalence, risk factors, and help-seeking behavior in opioid agonist treatment and general psychiatry: a cross-sectional study

**DOI:** 10.3389/fpsyt.2023.1204236

**Published:** 2023-08-07

**Authors:** Maximilian Meyer, Jean N. Westenberg, Patrick Brunner, Martin Gürtler, Gerhard A. Wiesbeck, Undine E. Lang, Marc Vogel, Kenneth M. Dürsteler

**Affiliations:** ^1^Clinic for Adult Psychiatry, University Psychiatric Clinics, University of Basel, Basel, Switzerland; ^2^Department of Psychiatry, University of British Columbia, Vancouver, BC, Canada; ^3^Health Center Allschwil (Gesundheitszentrum Allschwil AG), Allschwil, Switzerland; ^4^Department of Clinical Research, University of Basel, Basel, Switzerland; ^5^Department for Psychiatry, Psychotherapy and Psychosomatics, Psychiatric Hospital, University of Zurich, Zurich, Switzerland

**Keywords:** erectile dysfunction, sexual dysfunction, opioid maintenance, general psychiatry, opioid use disorder

## Abstract

**Background:**

Mental disorders pose a high risk for the occurrence of sexual dysfunctions (SD). This study aimed to investigate prevalence of risk factors and help-seeking behavior for sexual dysfunctions in patients with opioid use disorder compared to patients seeking psychotherapeutic help.

**Methods:**

Ninety-seven patients at two opioid agonist treatment (OAT) centers and 65 psychotherapeutic patients from a psychiatric practice (PP) in Switzerland were included in the study. Self-report assessments comprised sexual functioning (IIEF: International Index of Erectile Function; FSFI: Female Sexual Function Index), depressive state, psychological distress, alcohol consumption, nicotine use, and a self-designed questionnaire on help-seeking behavior. We used chi-squared and Mann–Whitney U tests for group comparisons and binary logistic regression models to identify variables predicting the occurrence of sexual dysfunctions.

**Results:**

There was no statistically significant difference (*p* = 0.140) in the prevalence of SD between OAT (*n* = 64, 66.0%) and PP sample (*n* = 35, 53.8%). OAT patients scored significantly higher in scales assessing nicotine use (*p* < 0.001) and depressive state (*p* = 0.005). Male OAT patients scored significantly worse on the Erectile Function scale (*p* = 0.005) and female PP patients scored significantly worse on the FSFI Pain domain (*p* = 0.022). Opioid use disorder, higher age, and being female predicted the occurrence of SD in the total sample. In the OAT sample, only higher age remained predictive for the occurrence of SD. A lack of help-seeking behavior was observed in both groups, with only 31% of OAT patients and 35% of PP patients ever having talked about their sexual health with their treating physician.

**Conclusion:**

SD are common among psychiatric patients receiving OAT and general psychiatric patients seeking psychotherapy. Professionals providing mental healthcare to patients must emphasize prevention and routine assessments of sexual functioning needs.

## 1. Introduction

Sexual functioning is an essential component of life and a significant contributor to quality of life and couple satisfaction (1). According to the Diagnostic and Statistical Manual of Mental Disorders (DSM-5, p. 423), “sexual dysfunctions are a heterogeneous group of disorders that are typically characterized by a clinically significant disturbance in a person’s ability to respond sexually or to experience sexual pleasure” (2). Whereas sexual dysfunctions (SD) in women mostly consist of reduced sexual desire, arousal, and orgasmic disorder, the most common SD in men are erectile dysfunction (ED) and premature ejaculation (3).

A broad range of risk factors for the occurrence of SD has been identified to date, with mental disorders being among the most important (3, 4). Indeed, some literature suggests the impact of poor mental health on sexual functioning is stronger than the impact of physical health problems (5). The link between substance use and SD is of particular interest, as elevated rates of impaired sexual functioning have been observed in individuals who use opioids. The prevalence of SD in opioid-dependent populations have been reported to be as high as 57 and 93% in women and men, respectively (6, 7). Comparable rates have been found in patients suffering from schizophrenia (8), obsessive–compulsive disorder (9), anxiety disorders (4), and depressive disorders (10). Whereas the link between antipsychotic and antidepressant medication and treatment-emergent SD has been well established, the link is less clear for the treatment of opioid use disorder (OUD). There is some evidence for opioid agonist treatment (OAT) improving male sexual functioning with stronger effects being observed in buprenorphine when compared to methadone (11, 12). However, no difference in SD prevalence has been observed in heroin-assisted treatment when compared to other forms of OAT (13).

Among patients with OUD, it remains unclear whether SD are caused by opioid intake itself or result from other substance-use related lifestyle factors (14, 15). Additionally, available literature linking alcohol and nicotine use to SD has been partly inconclusive. Moderate alcohol consumption may have a protective effect on ED (16, 17), whereas alcohol consumption at lower and high doses was reported to be predictive for ED (18, 19). Contradictory findings have also been reported for female SD. Moderate alcohol consumption was found to have a protective effect on hyposexuality in a cohort of Brazilian women (20), although a meta-review reported that in the majority of included studies, alcohol did not influence female sexual functioning at all (21). Regarding the use of nicotine, the available data is equally conflicting. It was demonstrated that under the influence of nicotine, erectile responses to erotic stimuli are substantially diminished, suggesting a dysfunction in the physiological mechanism of sexual arousal (22). Furthermore, the odds ratio for ED is 1.51 (95% CI: 1.34–1.71) for current tobacco smokers (23). Equally, some scholars reported a negative correlation for cumulative smoking dose and female sexual functioning (24), whereas McCool-Myers et al. found smoking to be a protective factor for female hyposexuality (21).

The marked impact that SD exert on the personal well-being is further aggravated by the fact that only a small fraction of affected individuals seek professional help (25). This could be counteracted by consistent sexual history taking by health care providers. However, physicians do not often take sexual history from their patients. One study reported the rate of sexual history taking among general practitioners to be as low as 15.5% (26), whereas the rate of exhaustive explorations in primary care has been found to be 1.1% (27).

In summary, individuals with OUD are at high risk of impaired sexual functioning, similar to other mental disorders. Moreover, SD are likely undertreated in opioid-dependent populations, as there is a lack of help-seeking behavior and sexual health is often not inquired about by treating physicians. Whereas risk factors like cigarette smoking and alcohol consumption on SD have been investigated, far less research has explored SD prevalence, SD risk factors, and SD help-seeking behavior in patients receiving OAT. Moreover, although recent studies have compared sexual desire and sexual dysfunction between men receiving methadone and buprenorphine maintenance treatment (28, 29), no study has directly compared SD between individuals receiving OAT and individuals receiving psychotherapeutic treatment for non-opioid-related mental health problems.

This study investigates SD in patients receiving OAT compared to patients with mental health problems seeking psychotherapeutic treatment. The research question was whether prevalence, contributing factors of SD, and help-seeking behaviors differed between patients with OUD recruited from two OAT centers and patients seeking psychotherapeutic treatment recruited from a psychiatric practice (PP).

## 2. Methods

### 2.1. Study settings and participants

Two patient populations, both of which were recruited in Switzerland, participated in the study. Patients with OUD were recruited from two outpatient treatment centers providing OAT. One center provided heroin-assisted treatment whereas the other center provided traditional OAT (methadone, slow-release oral morphine, and buprenorphine). The other patient population consisted of individuals recruited from a PP that offers psychotherapeutic treatment for a variety of mental health problems (non-opioid-related).

Individuals meeting the inclusion criteria had to be aged between 18 and 65 years and have sufficient German language skills to understand and complete questionnaires. Exclusion criteria were the presence of a medical condition or intake of pharmaceuticals which might affect their sexual functioning. Patients recruited at the PP who received any kind of psychopharmacological treatment, used substances besides alcohol or nicotine at the time of study conduction, or primarily sought help for the treatment of SD were excluded as well. A separate study on the OAT sample specifically investigating the differences between patients receiving heroin-assisted treatment and patients receiving traditional OAT has been published previously by the research group (13). All patients seeking care at one of the two outpatient treatment centers or at the psychiatric practice and who met the inclusion criteria were asked to participate in the study.

Recruitment took place between 2012 and 2016. A total of 169 patients participated in the study: 104 were recruited from OAT centers and 65 were recruited from PP. The sample size was derived from comparable studies in the field (12, 14, 30–32).

### 2.2. Measures

Sociodemographic characteristics (age, sex, housing situation, civil status, education, and employment status) were collected for each participant. Participants completed a battery of standardized self-report instruments, assessing sexual functioning, depressive state, psychological distress, alcohol use, and nicotine use. While completing the questionnaires all participants were able to ask clarifying questions.

The Female Sexual Function Index (FSFI) consists of 19 items assessing six domains of female sexual functioning (desire, arousal, lubrication, orgasm, satisfaction, and pain) over the past 4 weeks. Each item is rated on a 5-point Likert scale with higher scores indicating better sexual functioning (33). The cut-off score for differentiating women with and without SD is 26.55 with a sensitivity of 0.733 and a specificity of 0.889 (34).

The International Index of Erectile Function (IIEF) consists of 15 items assessing five domains of male sexual functioning (erectile function, orgasmic function, sexual desire, intercourse satisfaction, and overall satisfaction). Items are rated on a 5-point Likert scale (35). The short scale which includes erectile function-items only (IIEF-EF) has been validated as a diagnostic tool for ED with a cut-off score of 21 or lower indicating ED (sensitivity 0.89, specificity 0.93) (36).

The Allgemeine Depressionsskala (ADS-L) is the German adaption of the Center of Epidemiologic Studies - Depression Scale (CES-D) (37). This 20-item instrument utilizes a 4-point Likert scale and is widely used to assess depressive state. Higher scores indicate more severe depressive state and a cut-off score of 16 discriminated well between psychiatric patients and the general population (38).

The Alcohol Use Disorders Identification Test – Consumption (AUDIT-C) is a 3-item scale used to assess unhealthy alcohol use and the short version of the AUDIT (39). Each item is rated on a 5-point Likert scale. The cut-off score is ≥3 for women (sensitivity: 0.73, specificity: 0.91) and ≥ 4 for men (sensitivity: 0.86, specificity: 0.89) (40).

The Fagerström-Test for Nicotine Dependence (FTND-G) is a 6-item scale assessing nicotine dependence in relation to cigarette smoking. Items are summed up to a total score between 0 and 10. A score of 6 points or higher indicates ‘strong dependence’ (6–7 points) or ‘very strong dependence’ (≥ 8) (41).

The Symptom Checklist-27 (SCL-27) is the short version of the Symptom Checklist-90-R and is used to assess six psychological symptom domains (depressive, dysthymic, vegetative, agoraphobic, social phobia, and mistrust) (42). All items are rated on a 5-point Likert scale. The SCL-27 allows for the precise estimation of the Global Severity Index (GSI), which is a global composite score of overall psychological distress. A cut-off score of 0.5 showed the best psychometric properties (sensitivity: 0.83, specificity: 0.80) at discriminating psychiatric patients from a reference sample (43).

Lastly, participants completed a self-designed questionnaire about the role of sexual health during their contact with health care providers. The items inquired about whether participants themselves had ever sought help, whether they had ever been approached by their treating physician, and whether they had ever wished to receive counselling regarding their sexual health. Questions were answered on 5-point Likert scales and in yes/no form.

### 2.3. Statistical analysis

All data were analyzed with SPSS version 28 (IBM). Participants with missing data in one or more FSFI item or IIEF-EF item were excluded from the analysis (*n* = 7). Group comparisons were conducted with chi-squared tests for binary data and Mann–Whitney U tests for ordinal and continuous data. Binary logistic regression models were calculated to explore variables predicting SD. Level of significance was set at *p* = 0.05 for all calculations.

### 2.4. Ethics approval and consent to participate

This study was conducted in accordance with the Declaration of Helsinki and the study protocol was approved by the local ethics committee (Ethikkommission beider Basel, approval number: EK 31/11). All participants provided written informed consent.

## 3. Results

All participants were Caucasian and cisgender. Sociodemographic characteristics of the sample are provided in Table 1.

**Table 1:** Sample characteristics.

Characteristics; *n* (%)		OAT (*n* = 97)	PP (*n* = 65)	Total (*N* = 162)
Age; mean (SD)		41.9 (8.2)	43.4 (11.1)	42.5 (9.5)
Sex				
	Female	35 (36.1%)	28 (43.1%)	63 (38.9%)
	Male	62 (63.9%)	37 (56.9%)	99 (61.1%)
Ethnicity (Caucasian)		97 (100%)	65 (100%)	162 (100%)
Professional qualification		63 (64.9%)	55 (84.6%)	118 (72.8%)
Employment				
	Employed	14 (14.5%)	38 (58.5%)	52 (32.1%)
	Unemployed	33 (34.1%)	19 (29.2%)	52 (32.1%)
	Invalidity insurance	47 (48.5%)	8 (12.3%)	55 (34.0%)
	Missing	3 (3.1%)	-	3 (1.9%)
Housing				
	Alone	61 (62.9%)	21 (32.3%)	82 (50.6%)
	With partner	15 (15.5%)	38 (58.5%)	53 (32.7%)
	With parents	8 (8.2%)	3 (4.6%)	11 (6.8%)
	Shared apartment	3 (3.1%)	3 (4.6%)	6 (3.7%)
	Assisted living	7 (7.2%)	-	7 (4.3%)
	Missing	3 (3.1%)	-	3 (1.9%)
Civil status				
	Unmarried	77 (79.4%)	23 (35.4%)	100 (61.7%)
	Married	2 (2.1%)	24 (36.9%)	26 (16.0%)
	Divorced	12 (12.4%)	13 (20.0%)	25 (15.4%)
	Separated	2 (2.1%)	4 (6.2%)	6 (3.7%)
	Widowed	1 (1.0%)	1 (1.5%)	2 (1.2%)
	Missing	3 (3.1%)	-	3 (1.9%)

SD: standard deviation; Invalidity insurance: receiving rent because of medical disability.

### 3.1. Prevalence of sexual dysfunction

Occurrence of SD was defined as IIEF-EF score equal or lower than 21 for males (36) and an FSFI score equal or lower than 26.55 for females (34). Prevalence of SD was not significantly different between OAT patients (*n* = 64; 66.0%) and PP patients (*n* = 35; 53.8%), as determined by the chi-squared test [X^2^ (1, *n* = 162) = 2.4; *p* = 0.140]. SD occurred significantly more often in female patients (*n* = 52; 82.5%) than in male patients (*n* = 47; 47.5%) [X^2^(1, *n* = 162) = 19.9, *p* < 0.001].

### 3.2. Psychological distress, depressive state, nicotine use, and alcohol use

OAT patients scored significantly higher in the FTND (U = 1581.0, *p* < 0.001, r = 0.43) and the ADS-L (U = 2339.5, *p* = 0.005, r = 0.22) as determined by Mann–Whitney U tests (Table 2). No significant differences were found in total AUDIT-C scores and psychological distress as measured by the GSI. Mean scores and standard deviations of the scales are provided in Table 3.

**Table 2:** Female (FSFI domains) and male (IIEF-EF) sexual functioning among patients receiving OAT and patients in psychiatric practice.

FSFI Domain	Female patients receiving OAT (*n* = 35)		Female patients in PP (*n* = 28)		Mann–Whitney U	*p*-value	r
	Mean (SD)	Median (Min-Max)	Mean (SD)	Median (Min-Max)			
Desire	2.66 (1.47)	2.40 (1.20–5.40)	2.31 (1.43)	1.80 (1.20–6.00)	419.5	0.314	0.13
Arousal	2.50 (2.09)	2.10 (0.00–5.70)	1.95 (2.02)	1.05 (0.00–5.70)	404.5	0.234	0.15
Lubrication	2.59 (2.20)	3.00 (0.00–6.00)	2.22 (2.36)	1.50 (0.00–6.00)	444.0	0.516	0.08
Orgasm	2.13 (1.92)	2.40 (0.00–6.00)	2.39 (2.23)	2.40 (0.00–6.00)	450.0	0.570	0.07
Satisfaction	2.95 (1.64)	2.40 (0.00–6.00)	2.96 (2.13)	2.40 (0.80–6.00)	461.0	0.686	0.05
Pain	2.96 (2.57)	3.20 (0.00–6.00)	1.08 (2.10)	0.00 (0.00–6.00)	333.5	0.022*	0.29
IIEF	Male patients receiving OAT (*n* = 62)		Male patients in PP (*n* = 37)		Mann–Whitney U	*p*-value	r
Erectile Function	16.11 (11.02)	13.50 (1–30)	22.49 (8.89)	27.00 (2.00–30.00)	756.5	0.005**	0.28

SD: standard deviation; r: effect size.

* Indicating significance <0.05.

** Indicating significance <0.01.

**Table 3:** High-risk alcohol use (AUDIT-C), psychological distress (GSI), high-risk nicotine use (FTND), and depressive states (ADS-L) among patients receiving OAT and patients in psychiatric practice.

Scale	Patients receiving OAT (*n* = 97)		Patients in PP (*n* = 65)		Mann–Whitney U	*p*	r
	Mean (SD)	Median (Min-Max)	Mean (SD)	Median (Min-Max)			
AUDIT-C	3.45 (3.12)	3.00 (0.00–12.00)	2.88 (2.04)	3.00 (0.00–8.00)	2992.5	0.580	0.04
GSI	0.99 (0.83)	0.73 (0.00–3.38)	0.96 (0.71)	0.73 (0.00–2.87)	3086.5	0.681	0.02
FTND	4.99 (2.63)	5.00 (0.00–10.00)	2.37 (3.07)	0.00 (0.00–10.00)	1581.0	<0.001***	0.43
ADS-L	19.18 (9.80)	19.08 (0.00–44.00)	24.48 (11.62)	24.00 (6.00–51.00)	2339.5	0.005**	0.22

AUDIT-C: alcohol use disorders identification test-consumption; GSI: global severity index; FTND: Fagerström-test for nicotine dependence; ADS-L: Allgemeine Depressionsskala-Lang; SD: standard deviation; r: effect size.

** Indicates significance <0.01.

*** Indicates significance <0.001.

### 3.3. Factors contributing to the occurrence of sexual dysfunctions

Exploratory binary logistic regression was performed to identify predictors of SD in our study population. Variables included were age, sex, whether patients had OUD, depressive state as measured by the ADS-L, overall psychological distress as measured by the GSI, high-risk use of alcohol as measured by the AUDIT-C, and nicotine dependence as measured by the FTND. Being female (*p* < 0.001), of higher age (*p* = 0.008), and having OUD (*p* = 0.024) were predictive for the occurrence of SD in the total sample (Table 4).

**Table 4:** Logistic regression on SD prevalence in the total sample (*N* = 162).

Variable	B	*p*	OR	CI (95%)
Psychological distress	0.024	0.944	1.025	0.519–2.022
Alcohol use	−0.051	0.462	0.951	0.831–1.088
Nicotine use	0.044	0.518	1.045	0.914–1.194
Depressive state	0.043	0.084	1.044	0.994–1.097
OUD	1.000	0.024*	2.718	1.140–6.484
Sex^a^	1.551	<0.001***	4.717	2.035–10.933
Age	0.054	0.008**	1.056	1.014–1.098

Nagelkerkes R^2^ = 0.274; Hosmer-Lemeshow test: X^2^ = 7.460, *p* = 0.488. OR: odds ratio; CI: confidence interval.

* Indicates significance < 0.05.

** Indicates significance < 0.01.

*** Indicates significance < 0.001.

a Female sex was the reference category.

To find out whether predictive variables differed within each respective group, calculations of logistic regression models were repeated for the OAT and PP sample, respectively. The opioid dependence variable was not included in these models. In the OAT sample, higher age (B = 0.072; *p* = 0.015; OR = 1.074) remained a significant predictor of SD. In the PP sample, being female (B = 1.961; *p* < 0.004; OR = 7.110) significantly predicted the presence of SD.

### 3.4. Sexual functioning related help-seeking behavior

The results of the self-designed questionnaire used to assess self-perceived importance of sexual functioning and related help-seeking behavior are presented in Table 5. We found no differences between the OAT and the PP patients as determined by the Mann–Whitney U test in questions 1 to 5. Similarly, no differences were found between groups regarding whether patients had ever talked about their sexual health to their treating physician [X^2^ (1, *n* = 162) = 0.351; *p* = 0.610] and whether their sexual health had ever been inquired about by their treating physician [X^2^ (1, *n* = 162) = 0.994; *p* = 0.366] as determined by the chi-squared test.

**Table 5:** Descriptive results of the questionnaire on sexual functioning and related help-seeking behavior.

Survey questions	OAT (n = 97)	PP (n = 65)	Total (n = 162)
1. How important is sexual satisfaction for your general well-being? (0–4); mean (SD)	2.46 (1.35)	2.31 (1.17)	2.40 (1.28)
2. How important is sexual functioning (arousal, orgasm) for your general well-being? (0–4); mean (SD)	2.64 (1.30)	2.37 (1.15)	2.53 (1.25)
3. How important is sexual functioning for your sexual satisfaction? (0–4); mean (SD)	2.60 (1.22)	2.48 (1.11)	2.55 (1.18)
4. Do you think your treating physicians consider the influence on your sexuality when choosing their medication? (0–4); mean (SD)	1.68 (1.23)	1.95 (1.07)	1.79 (1.17)
5. Have you ever wanted to receive counselling regarding your sexual health by your treating physician? (0–4); mean (SD)	1.26 (1.28)	1.06 (1.17)	1.18 (1.24)
6. Have you ever talked to your treating doctors about your sexual health and related issues? (yes/no); n (%)	30 (30.9%)	23 (35.4%)	53 (31.4%)
7. Has your treating physician ever inquired about your sexual health and sexual satisfaction on their own initiative? (yes/no); n (%)	23 (23.7%)	20 (30.8%)	44 (26.0%)

Questions 1–5 were rated on 5-point Likert-scales (0 = no/not important; 4 = yes/very important); Questions 6–7 were asked in yes/no-form.

Help-seeking behavior differed significantly by SD status. Patients suffering from SD were significantly less likely to have ever talked to their treating physicians about their sexual health (*n* = 26, 26.2%) when compared to patients without SD (*n* = 27, 42.9%) [X^2^ (1, *n* = 162) = 4.816; *p* = 0.039]. No difference regarding the wish to receive counselling (question 5) was found between patients with and without SD as determined by the Mann–Whitney U test.

## 4. Discussion

This study examined the prevalence of SD in a sample of patients receiving OAT and a sample of patients seeking psychotherapy from a general psychiatric practice. We found a higher prevalence of SD in the OAT sample (66.0%) when compared to the PP sample (53.8%), with the difference not being statistically significant. This result is likely masked due to the unequal sex distribution in our sample. Only 36.1% of OAT patients were female compared to 43.1% of PP patients, with the latter showing worse sexual health and scoring significantly worse on the Pain domain of the FSFI. Furthermore, female sex remained a significant predictor of SD in PP patients, which was not the case for OAT patients. This observation could explain why, when controlling for sex in the logistic regression model of the total sample, presence of OUD predicted the occurrence of SD. Therefore, even though no significant differences in prevalence were found, the regression model demonstrates that OUD poses a risk for developing SD when compared to milder psychiatric disorders. In line with this, patients with mild transient physically-related illnesses have been found to have a significantly lower prevalence of SDs when compared to patients with OUD receiving OAT (13). A study comparing psychiatric patients to physically ill patients found significantly higher rates of SD in the psychiatric sample, adding to the existing evidence for the impact of mental health on sexual functioning (43).

When specific sexual functioning domains were compared in our study, men in the OAT sample scored significantly lower in the Erectile Function domain of the IIEF whereas women in the PP sample scored significantly lower in the Pain domain of the FSFI, both indicative of worse overall sexual functioning. This highlights the need for more research on specific sexual dysfunctions within different psychiatric subpopulations (e.g., pain in women), in order to provide better individualized and targeted treatment strategies to patients suffering from SD. Other differences were identified between the samples, such as significantly higher FTND and ADS-L scores (assessments for nicotine use and depressive symptoms respectively) among those receiving OAT when compared to the PP sample, which may have influenced the presence or absence of specific sexual dysfunctions in the unique samples. However, in our sample, both nicotine use and depressive state did not significantly predict SD in isolation.

Presence of OUD, higher age, and being female predicted the occurrence of SD in the total sample. In the logistic regression analysis, higher age was a predictor of SD among individuals receiving OAT but not in the general PP sample. This might be related to the hormonal effects of prolonged opioid use, continued use of other substances (e.g., cocaine), as well as prolonged presence of substance use-related lifestyle factors (e.g., poor nutrition, impaired physical health, reduced fitness) (44). However, this is speculative, as this study did not control for these variables. Nonetheless, the influence of higher age is an important finding. As the opioid-dependent population is ageing in Switzerland and European countries, individuals receiving OAT are likely to be at even greater risk of developing SD in the future (45). Surprisingly, depressive state was not a predictor of SD in neither of the samples, despite recent literature demonstrating high rates of SD in populations suffering from depressive disorders (10). As for the impact of alcohol and nicotine use on SD, the available literature has been partly inconclusive (16, 17, 21, 24), and our results unfortunately do not provide more clarity. In our study, neither nicotine use nor alcohol use predicted the occurrence of SD in the logistic regression models, despite a significant difference in FTND scores being observed between the PP and OAT sample.

Regarding help-seeking behavior, no significant differences were found between the PP and the OAT sample. Although we observed a high prevalence of SD in both samples, only 31% of OAT patients and 35% of PP patients had ever talked about sexual health issues with their treating physician. Interestingly, both patient populations seemed to not want to receive counselling regarding their sexual health from their treating physician. This is an important finding, as SD appear to be particularly common in psychiatric patients (4). When compared by SD status, patients suffering from SD w. This is problematic, as help-seeking behavior seems to be lowest in patients who would likely benefit the most from counselling. Future studies should explore the underlying reasons behind these observations in order to best address possible stigma and perceived barriers as it relates to sexual function/dysfunction. For instance, patients may feel generally uncomfortable talking about their sexual health with their treating physician or may believe their treating physician would not be able to help them and may prefer talking to a sexual health specialist instead.

### 4.1. Limitations

This study has several limitations. First, even though similar prevalence rates of SD have been observed in a variety of mental disorders, the specific problem/mental disorder that patients in the control sample sought psychotherapeutic help for was not collected and therefore not controlled for in our analysis. Second, the convenience sampling, the moderate sample size, and the unequal gender distribution limit the generalizability of the presented findings. Third, we did not control for opioid dose, other medication, duration of illness, as well as mental and physical comorbidities Future studies should systematically collect and control for these variables in order to determine their impact on sexual functioning among patients receiving OAT.

The representative samples of two distinct psychiatric populations in clinical settings (i.e., individuals seeking help for milder psychiatric problems and the opioid-dependent population) is a notable strength of our study.

## 5. Conclusion

SD are common among psychiatric patients receiving OAT and general psychiatric patients seeking psychotherapy, with higher age, female sex, and presence of OUD predicting SD. Future research should focus on specific sexual dysfunctions (e.g., pain in women), within different psychiatric subpopulations, in order to provide better individualized and targeted treatment strategies to patients suffering from SD. Despite low rates of help-seeking and surprisingly low rates of perceived need for sexual functioning healthcare, professionals providing mental healthcare to patients must emphasize prevention and routine assessments of sexual functioning needs.

## Data availability statement

The raw data supporting the conclusions of this article will be made available by the authors, without undue reservation.

## Ethics statement

The studies involving human participants were reviewed and approved by Ethikkommission beider Basel. The patients/participants provided their written informed consent to participate in this study.

## Author contributions

MM was involved in data curation and formal analysis. MM and JW wrote the original draft. PB and MG were involved in investigation. GW and UL contributed to writing: review and editing, and resources. MV and KD supervised the project. KD was involved in conceptualization, methodology, and writing: review and editing. All authors contributed to the article and approved the submitted version.

## Conflict of interest

The authors declare that the research was conducted in the absence of any commercial or financial relationships that could be construed as a potential conflict of interest.

## Publisher’s note

All claims expressed in this article are solely those of the authors and do not necessarily represent those of their affiliated organizations, or those of the publisher, the editors and the reviewers. Any product that may be evaluated in this article, or claim that may be made by its manufacturer, is not guaranteed or endorsed by the publisher.
